# Neurochemistry of Enteric Neurons Following Prolonged Indomethacin Administration in the Porcine Duodenum

**DOI:** 10.3389/fphar.2020.564457

**Published:** 2020-09-08

**Authors:** Marta Czajkowska, Jarosław Całka

**Affiliations:** Department of Clinical Physiology, Faculty of Veterinary Medicine, University of Warmia and Mazury, Olsztyn, Poland

**Keywords:** pig, duodenum, nonsteroidal antiinflammatory drugs, enteric nervous system, neuronal plasticity

## Abstract

Gastrointestinal inflammation resulting from prolonged NSAID drugs treatment constitutes a worldwide medical problem. The role of enteric neuroactive substances involved in this process has recently gained attention and neuropeptides produced by the enteric nervous system may play an important role in the modulation of gastrointestinal inflammation. Therefore, the aim of this study was to determine the effect of inflammation caused by indomethacin supplementation on vasoactive intestinal polypeptide (VIP), substance P (SP), neuronal nitric oxide synthase (nNOS), galanin (GAL), pituitary adenylate cyclase-activating polypeptide (PACAP), and cocaine- and amphetamine-regulated transcript peptide (CART) expression in enteric duodenal neurons in domestic pigs. Eight immature pigs of the Pietrain × Duroc race (20 kg of body weight) were used. Control animals (n=4) received empty gelatine capsules. Experimental pigs (n=4) were given indomethacin for 4 weeks, orally 10 mg/kg daily, approximately 1 h before feeding. The animals from both groups were then euthanized. Frozen sections were prepared from the collected duodenum and subjected to double immunofluorescence staining. Primary antibodies against neuronal marker PGP 9.5 and VIP, nNOS, SP, GAL, CART, and PACAP were visualized with Alexa Fluor 488 and 546. Sections were analyzed under an Olympus BX51 fluorescence microscope. Microscopic analysis showed significant increases in the number of nNOS-, VIP-, SP-, GAL-, PACAP-, and CART-immunoreactive ganglionic neurons, in both the myenteric and submucous plexuses of the porcine duodenum. The obtained results show the participation of enteric neurotransmitters in the neuronal duodenal response to indomethacin-induced inflammation.

## Introduction

Nonsteroidal antiinflammatory drugs (NSAIDs), which include indomethacin, lead to the development of ulcer lesions in both humans and laboratory animals as a result of long-term administration. Many factors are involved in the pathomechanism of these changes such as bile acid, bacterial flora and nitric oxide (NO). The most important role in the formation of enteropathy induced by NSAIDs supplementation is the deficiency of endogenous prostaglandins (Wallace, 2013).

The most commonly used NSAIDs, including indomethacin, act by inhibiting cyclooxygenase (COX). The inhibition of COX-1, the constitutive form, is responsible for the ulcerogenic effect. COX-1 occurs under physiological conditions (platelets, gastric mucosa, kidneys, endothelium) and is involved in the transformation of arachidonic acid into prostaglandin E2, I2 and thromboxane A2. Therefore, it acts cytoprotectively on the gastrointestinal tract, has a positive effect on the blood flow and regulates the activity of platelets (Vane and Botting, 1998). Inhibition of COX-2, an inducible form of the enzyme, is not directly related to the formation of mucosal damage of the gastrointestinal tract. Cyclooxygenase 2 is formed mainly due to the action of endotoxin or proinflammatory cytokines (IL-1, TNF-α), and the presence of COX-2 mRNA was found in inflamed tissues (Zarghi and Arfaei, 2011). COX-2 is responsible for the formation of prostaglandins inducing an increase in vascular permeability, edema and pain. However, it has been proven that both inhibition of COX-1 and COX-2 synthesis is crucial in the development of gastrointestinal disorders. This indicates that both isoforms of this enzyme are involved in the proper functioning of the stomach and intestines (Morita, 2002).

In order to prevent and eliminate changes, proton pump inhibitors (IPP) are recommended during co-administration with NSAIDs. These drugs effectively inhibit basic and stimulated gastric secretion (Scheiman, 2013). Long-term administration of IPP increases the possibility of the occurrence of serious side effects. Prolonged use of IPP results in a persistent increase in pH in the stomach, which stimulates the secretion of gastrin. Hypergastrinemia is associated with the possibility of ECL cell (enterochromaffin-like cells) hyperplasia, carcinoids and polyps (Jalving et al., 2003). Chronic hypochlorhydria, enduring the protective effect of hydrochloric acid on the growth of pathogenic bacteria, may lead to infectious complications of the lungs and gastrointestinal tract (Laine et al., 2000; de Jager et al., 2012). Since important adverse reactions are a significant obstacle to the long-term use of IPP, the identification of effective therapies for treating NSAID-induced gastrointestinal lesions remains an urgent priority.

The functions of the digestive tract are controlled by a network of neurons called the enteric nervous system (ENS). The ENS consists of myenteric plexus (MP) and submucosal plexus (SP). The latter are divided into two separate groups of plexuses: inner submucosal plexus (ISP), near the lamina propria of the mucosal layer and the outer submucosal plexus (OSP) adhering directly to the circular muscular layer (Bulc et al., 2019).

The enteric nervous system is involved in most physiological and pathophysiological processes in the gastrointestinal tract. ENS regulates the activity of the stomach and intestines, including peristalsis, secretion, blood flow and the immune system (Hansen, 2003). In recent years, there has been tremendous progress in understanding the pharmacological basis of the functioning of the ENS and its essential role in the functioning of the intestines in diseases. In fact, knowledge about ENS has provided an inspiration for the treatment of many gastrointestinal disorders. Several new therapeutic concepts are already in clinical use, such as serotonergic drugs in functional intestinal diseases (Tack, 2000; Wood, 2004).

ENS plays a role in dysfunctions such as peristaltic disorders leading to diarrhea or constipation, bowel ischemia and gastrointestinal inflammation (Tack, 2000). In response to numerous proinflammatory factors, ENS demonstrates neural plasticity, which is an adaptation of the nervous system through functional adjustment of neurons to changeable conditions. The release of neurotransmitters is involved in the formation and development of inflammatory changes as well as in regeneration processes.

Due to its anatomical and physiological resemblance to humans, the pig is considered the most valuable animal model used in gastroneuronal research. The similarities also apply to body size and composition. In addition, the pathomechanism of the development of gastroduodenal ulcers caused by stress and the administration with NSAIDs, in pigs and humans is similar (Rainsford et al., 2003).

Many authors indicate that the NSAIDs administration causes bowel disorders such as enterocyte injury, impaired absorption and increased intestinal permeability, diarrhea, bleeding, ulceration and perforation (Lomax et al., 2005; Bjarnason and Takeuchi, 2009). The influence of these drugs on macroscopic changes in the stomach and intestines has been examined. However, there are no reports suggesting the effect of indomethacin on the expression of neurotransmitters within the gastroduodenal region where inflammation usually occurs. Due to the involvement of ENS in the pathogenesis of gastrointestinal dysfunction, knowledge of the phenotypes of enteric duodenal neurons is necessary to determine the role of the studied neurotransmitters in the development of side effects of indomethacin treatment.

Considering the above, the main aim of this study was to determine the expression of VIP, nNOS, SP, GAL, PACAP, and CART in the porcine duodenal enteric neurons and to establish the effect of prolonged indomethacin supplementation on their neurochemical characteristics.

## Materials and Methods

The present experiment was performed on eight female piglets of the Pietrain × Duroc race (approximately 20 kg of body weight) obtained from a breeding farm in Lubawa (Poland). The animals were housed in cages for four animals each in a standardized environment at 20°C–22°C, 55%–60% relative humidity and 12 h/12 h light-dark cycle. The pigs were fed twice daily with a complete feed mix adapted to their needs and age. The animals were kept and treated in accordance with the rules approved by the Local Ethical Committee in Olsztyn (decision no. 54/2017 from 25 July 2017).

Immediately after the assimilation period lasting seven days, the animals were divided into two groups: control (C group) and experimental (I group). Group C (n=4) consisted of animals which received empty gelatine capsules. Group I (n=4) was composed of pigs which were given indomethacin orally (Metindol Retard; PharmaSwiss Česká republika s.r.o.; Republika Czeska; 10 mg/kg b. w.). The capsules were given to all animals in the same way—once a day, about 1 h before the morning feeding. Before starting indomethacin administration and once a week, pigs from research groups were weighed to determine the appropriate dose of the drug. In addition, the selection of only one sex of animals allowed excluding unknown sex-dependent changes in the chemical coding of porcine duodenal neurons.

After the indomethacin administration period lasting 28 days, all piglets were euthanized by premedication with Stresnil (Stresnil; Janssen Pharmaceutica N.V., Belgium, 2 mg/kg b. w., i.m.) and (after 20 min) an overdose of sodium thiopental (Thiopental, Sandoz, Kundl‐Rakúsko, Austria, i.v.). After confirming the cessation of vital functions, the material (3 cm fragments of duodenum located 10 cm caudal to musculus sphincter pylori) was taken for further analysis. The duodenal fragments were fixed in a solution of 4% buffered paraformaldehyde (pH 7.4) for 1 h. Afterwards, the tissues were inserted in three changes of phosphate buffer for 72 h. The test materials were then placed in an 18% sucrose solution for three weeks at 4°C. Fourteen-µm-thick cryostat sections of duodenal samples were used in double-labeling immunofluorescence (according to an earlier described method) (Czajkowska et al., 2020).

In the next stage, the prepared sections were dried for 45 min and then were washed in three changes of 0.1 M phosphate-buffered saline (PBS) for 15 min. The composition of the PBS solution was (in mM/l) 137 NaCl, 2.7 KCl, 10 NaH_2_PO_4_, and 1.8 KH_2_PO_4._ The duodenal samples were soaked for 1 h in the blocking mixture consisting of 10% horse serum and 0.1% bovine serum albumin in 0.1 M PBS, 1% Triton X-100, 0.05% Thimerosal, and 0.01% sodium azide in room conditions. After three washes in PBS, the sections were immunolabeled with protein gene-product 9.5 (PGP 9.5, used as a pan-neuronal marker). As primary antisera, vasoactive intestinal polypeptide (VIP), substance P (SP), neuronal nitric oxide synthase (nNOS), galanin (GAL), pituitary adenylate cyclase-activating polypeptide (PACAP), and cocaine- and amphetamine-regulated transcript peptide were used (CART). The overnight incubation with a mixture of primary antibodies was carried out under room conditions.

After 3 × 10 min of thorough washes in PBS, the tissues were incubated in secondary antibodies Alexa fluor 488 or 546 for 1 h. Primary and secondary antibodies, dilutions and sources are listed in **Table 1**. To assess the antibodies, as well as the specificity of the method, standard controls were used, which include pre-absorption of primary antisera with studied substances, omission and replacement of primary antisera by nonimmune sera.

**Table 1:** Primary and secondary antibodies used for immunohistochemistry experiments.

Antigen	Species	Dilution	Code	Supplier
PGP 9.5	Mouse	1:1,000	7863-2004	BioRad, Hercules, CA,USA
VIP	Rabbit	1:2,000	ab22736	Abcam, United Kingdom
SP	Rabbit	1:1,000	8450-0004	BioRad, Hercules, CA,USA
nNOS	Rabbit	1:3,000	AB5380	Chemicon, USA
GAL	Rabbit	1:2,000	4600-5004	Biogenesis, UK
PACAP	Guinea Pig	1:3,000	T-5039	Peninsula, San Carlos, CA, USA
CART	Rabbit	1:16,000	H-003-61	Phoenix Pharmaceuticals, Inc., Burlingame, CA, USA
Alexa fluor 488 donkey anti-mouse IgG		1:1,000	A21202	ThermoFisher Scientific, Waltham, MA, USA
Alexa fluor 546 donkey anti-rabbit IgG		1:1,000	A11010	ThermoFisher Scientific, Waltham, MA, USA
Alexa fluor 546 donkey anti-guinea pig IgG		1:1,000	A11074	ThermoFisher Scientific, Waltham, MA, USA

The tissues were examined with a fluorescence microscope (Olympus BX51) equipped with epifluorescence and appropriate filter sets. All images were acquired using a digital camera connected to a PC and processed with Olympus Cell F image-analysis software (Olympus, Tokyo, Japan).

To determine the percentages of particular neuronal populations, at least 500 of PGP 9.5-positive neurons were counted to specify the phenotype of the nerve cells in each enteric duodenal plexus (myenteric, inner and outer submucosal). Only neurons with a clearly visible cell nucleus were counted in the study. The sections to which the same antibody mixture was applied were separated by at least 100 μm to avoid double analysis of neuronal expression.

The results were presented as mean ± standard error. The statistical significance of differences between the study groups was defined using an independent sample t-test by Statistica 12 software (StatSoft Inc., Tulsa, OK, USA). A *p*-value of 0.05 or less was considered statistically significant.

## Results

Post-mortem analysis of collected materials showed pathological signs in pigs treated with indomethacin compared to control animals. On the duodenal mucosa of experimental pigs, inflammatory changes such as ecchymosis, hyperemia, edema and excessive mucus production were observed.

**Figure 1:**
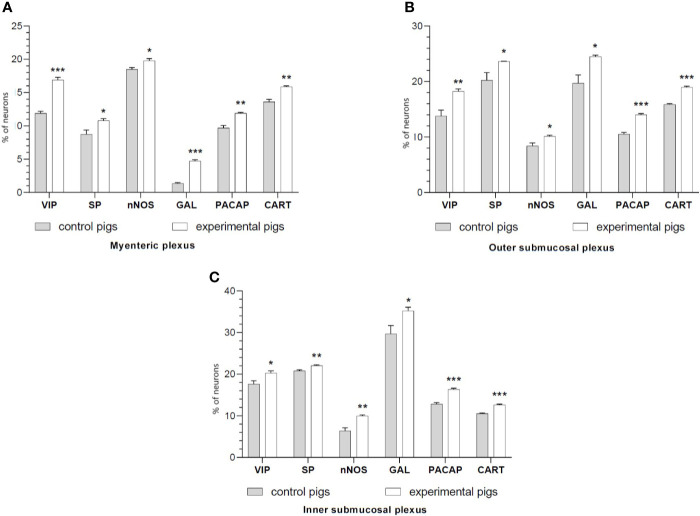
The graphs show the percentage of the neurons vasoactive intestinal polypeptide (VIP)-IR, substance P (SP)-IR, neuronal nitric oxide synthase (nNOS)-IR, galanin (GAL)-IR, pituitary adenylate cyclase-activating polypeptide (PACAP)-IR and cocaine- and amphetamine-regulated transcript (CART)-IR in the myenteric plexuses **(A)**, outer submucosal plexuses **(B)** and inner submucosal plexuses **(C)** in porcine duodenum in the control (gray bar) and experimental (white bar) groups. *p < 0.05, **p < 0.01, ***p < 0.001 indicates differences in the expression of individual test substances compared to control pigs.

**Figure 2:**
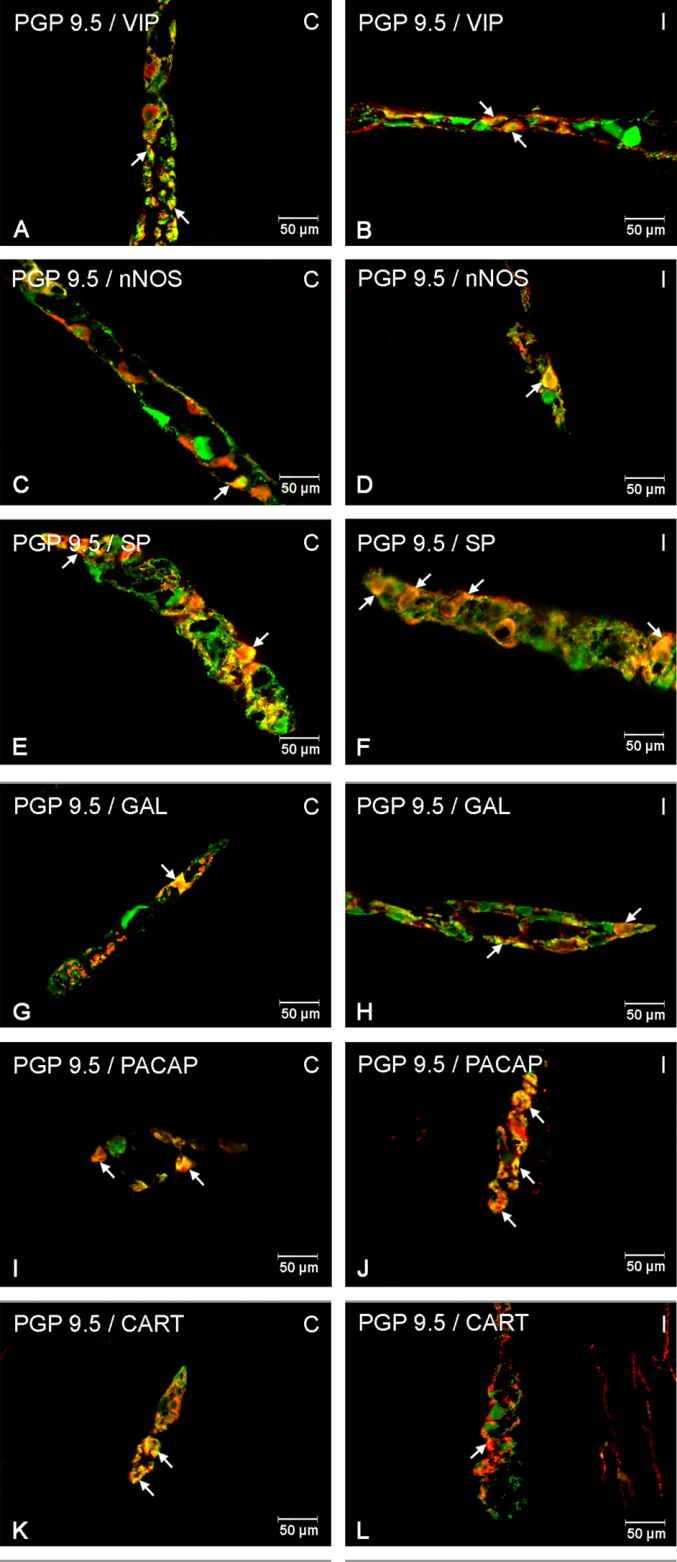
Enteric cells in porcine duodenal myenteric plexuses in physiological conditions **(C)** and after indomethacin administration **(I)**. Neurons containing the protein gene-product 9.5 (PGP 9.5) pan-neuronal marker (green) and other neuronal substances studied such as vasoactive intestinal polypeptide (VIP) **(A**, **B)**, neuronal nitric oxide synthase (nNOS) **(C**, **D)**, substance P (SP) **(E**, **F)**, galanin (GAL) **(G**, **H)**, pituitary adenylate cyclase-activating polypeptide (PACAP) **(I**, **J)**, and cocaine- and amphetamine-regulated transcript peptide (CART) **(K**, **L)** colocalized with PGP 9.5 (yellow/orange). The arrows in the photos show coexpression of PGP 9.5 and other test neurotransmitters.

**Figure 3:**
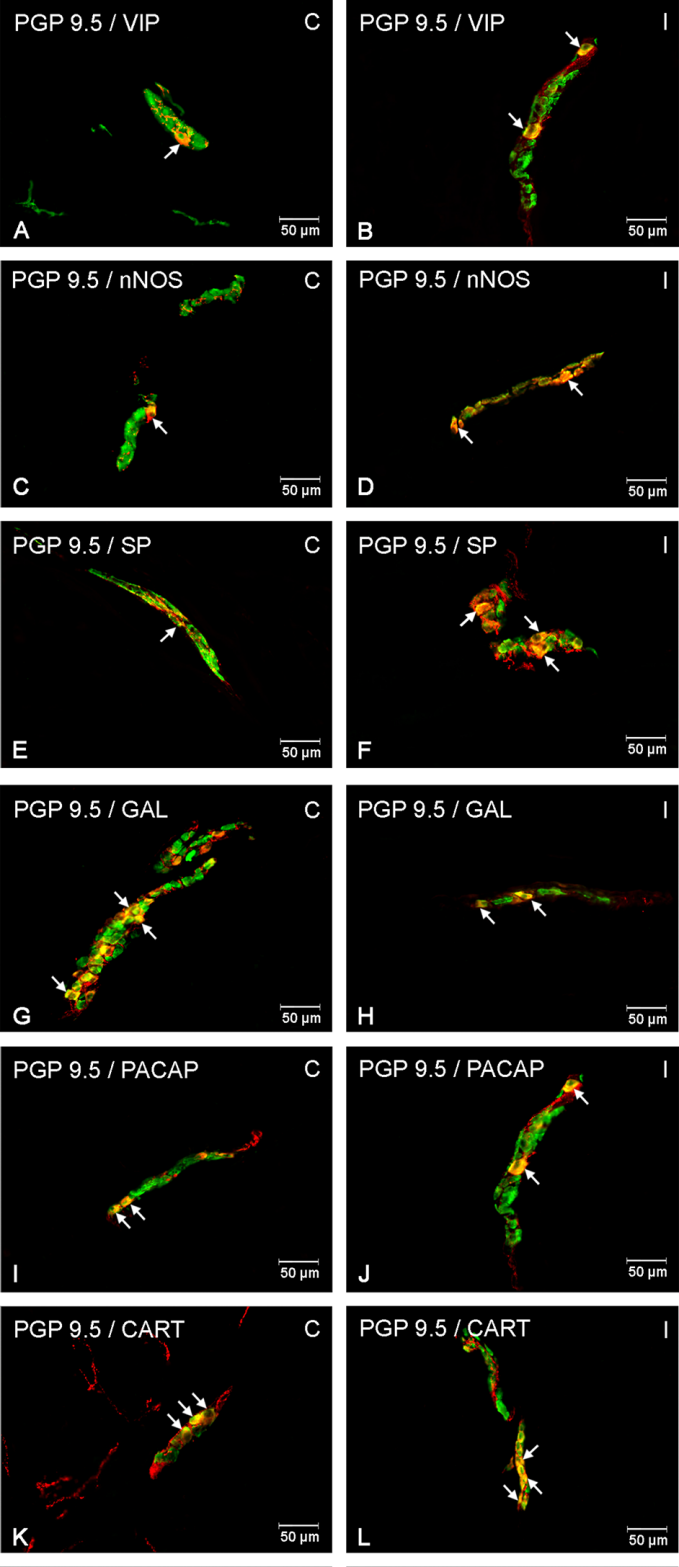
Enteric cells in porcine duodenal outer submucosal plexuses in physiological conditions **(C)** and after indomethacin administration **(I)**. Neurons containing the protein gene-product 9.5 (PGP 9.5) pan-neuronal marker (green) and other neuronal substances studied such as vasoactive intestinal polypeptide (VIP) **(A**, **B)**, neuronal nitric oxide synthase (nNOS) **(C**, **D)**, substance P (SP) **(E**, **F)**, galanin (GAL) **(G**, **H)**, pituitary adenylate cyclase-activating polypeptide (PACAP) **(I**, **J)** and cocaine- and amphetamine-regulated transcript peptide (CART) **(K**, **L)** colocalized with protein gene-product 9.5 (PGP 9.5) (yellow/orange). The arrows in the photos show co‐expression of PGP 9.5 and other test neurotransmitters.

**Figure 4:**
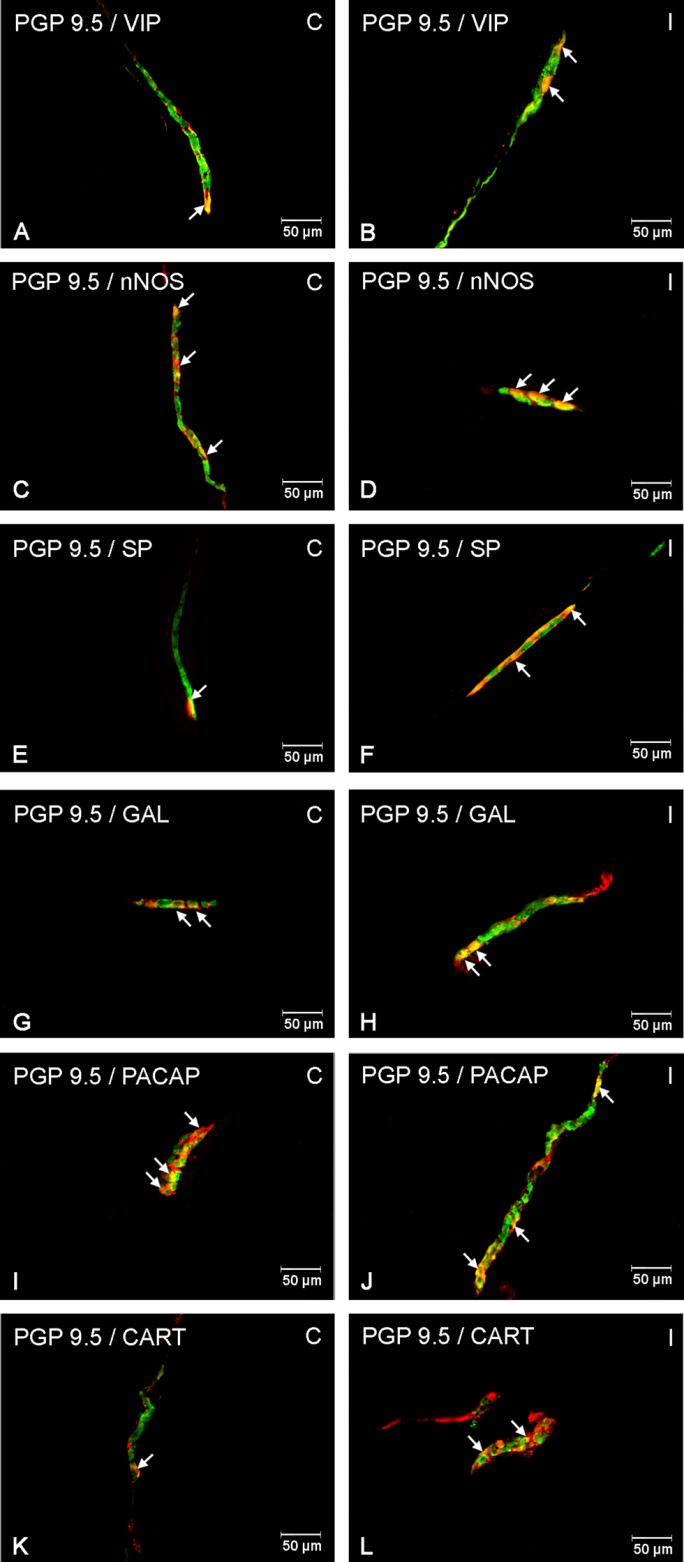
Enteric cells in porcine duodenal inner submucosal plexuses in physiological conditions **(C)** and after indomethacin administration **(I)**. Neurons containing the protein gene-product 9.5 (PGP 9.5) pan-neuronal marker (green) and other neuronal substances studied such as VIP **(A**, **B)**, nNOS **(C**, **D)**, SP **(E**, **F)**, GAL **(G**, **H)**, PACAP **(I**, **J)** and CART **(K**, **L)** colocalized with PGP 9.5 (yellow/orange). The arrows in the photos show co‐expression of PGP 9.5 and other test neurotransmitters.

It was noted that indomethacin changes the chemical coding of intramural duodenal neurons. This treatment resulted in a significant increase in the number of VIP-, SP-, nNOS-, GAL, PACAP- and CART-IR neurons in the three investigated enteric plexuses (**Figures 1**–**4**).

Double-labeling immunohistochemistry showed that the control duodenum MP, OSP and ISP possessed 11.9%, 13.78%, and 17.63% of PGP 9.5-positive neurons expressing VIP, respectively. Indomethacin treatment increased the numbers of the neurons to 16.91%, 18.24%, 20.28% in MP, OSP and ISP, respectively.

The number of nNOS-IR neurons in the control intestine amounted 18.50%, 8.40% and 6.39% of the PGP 9.5-IR nerve cells in MP, OSP and ISP, correspondingly. A significant increase in the number of nNOS-positive neurons to 19.79% in MP, 10.13% in OSP, and 9.99% in ISP was found in experimental animals.

SP expression was found in 8.74% (MP), 20.22% (OSP), 20.80% (ISP) of PGP 9.5-IR cells of the control duodenum, while in investigated animals it increased to 10.80% in MP, 23.62% in OSP and 22.07% in ISP.

In physiological conditions, the duodenum revealed 1.36%, 19.71%, 29.67% of GAL-IR cells in MP, OSP and ISP, respectively. Indomethacin supplementation resulted in an increased number of GAL-IR neurons in MP to 4.73%, OSP to 24.50% and ISP to 35.22%.

PACAP-positive duodenal intramural neurons in control animals constituted 9.69%, 10.51%, and 12.82% of the PGP 9.5-IR population of MP, OSP and ISP, respectively. However, indomethacin administration caused PACAP expression in an increased number of the enteric duodenal neurons, up to 11.91% in MP, 14.02% in OSP and 16.36% in ISP.

CART immunoreactivity was identified in 13.61%, 15.88%, 10.55% of control neurons in MP, OSP and ISP, respectively. The experimental procedure up-regulated the number of CART-IR nerve cells in MP, OSP and ISP to 15.89%, 18.96%, and 12.64%, respectively.

## Discussion

The upper part of the digestive tract is particularly exposed to irritating substances. In fact, the balance between protective and pathological factors affecting the duodenal mucosa plays a decisive role in the pathogenesis of mucosal damage. As demonstrated by many years of observation and numerous randomized clinical trials, NSAIDs play an important role in the pathogenesis of damage to the duodenal mucosa. 2%–19% of patients treated chronically with NSAIDs have duodenal ulcers (Blower, 1991).

To the best of the authors’ knowledge, this is the first study to demonstrate changes in the expression of neurotransmitters in duodenal pig neurons caused by treatment with indomethacin. The experiment revealed the increase of VIP-, SP-, nNOS-, GAL-, CART-, and PACAP-IR enteric neurons in MP, OSP and ISP in the examined intestine.

The available literature demonstrates that neuropeptides perform key regulatory functions in the development of gastrointestinal inflammation (Fernandes et al., 2009). The enteric nervous system is closely related to the immune system. In response to inflammation, ENS reacts by changing the expression of neurotransmitters and/or neuromodulators. The inflammatory effect caused by the administration of indomethacin led to phenotypic changes of porcine duodenal enteric cells (Furness et al., 2014). SP, which is the most important initiator of gastrointestinal lesions, is involved in the development of neurogenic inflammation.

SP expression was demonstrated in inflammatory conditions of the mucous membrane of the gastrointestinal tract. It is released from the enteric nerves, sensory neurons and inflammatory cells of the lamina propria. SP stimulates the secretion of cytokines, chemokines and other substances. These, in turn, modulate inflammation, motility and diarrhea, which are related to the pathophysiology of intestinal disorders (Koon and Pothoulakis, 2006). SP is released from axons exposed to damaging factors, and this response ultimately contributes to initialization of inflammatory cascades.

Inflammation-induced release of SP was observed in myenteric plexuses in rat colitis following dextran-sulfate supplementation (Kishimoto et al., 1994). Similar observations were noted in the stomach of acrylamide-treated pigs (Palus et al., 2018). Akbar et al. (2008) revealed an increase in the number of SP-positive nerve fibers in colon biopsies collected from patients with irritable bowel syndrome (IBS). In addition, SP has recently been shown to induce the secretion of proinflammatory cytokines by various cell types. The stimulation of human monocytes by SP caused the release of IL-1 (Laurenzi et al., 1990). It has been proven that substance P modulates IFN-gamma secretion *via* the NK-1 receptor (Blum et al., 1993). Administration of SP in an animal model led to the induction of mast cells, which consequently extended the synthesis of TNF-α and IL-6 through activation of NF-κB (Azzolina et al., 2003).

Galanin is also involved in gastrointestinal inflammatory reactions. In the digestive tract, GAL is noted in enteric cells as well as in fibers in all duodenal layers. It is a multifunctional substance and controls biological functions, such as secretion and intestinal contraction (Pham et al., 2002).

GAL plasticity within the enteric nervous system has been proven using various models of gastrointestinal damage, for example in the experimental model of formalin-induced colitis (Gonkowski et al., 2010). Enteric upregulation of galanin was observed in the digestive system after infection with intestinal bacteria naturally occurring in pigs (Pidsudko et al., 2008). According to studies of Simpson et al. (2009), an increase in galanin expression was noted in the enteric nervous system in patients with diverticulitis (Simpson et al., 2009). The presented studies are able to correlate with these observations.

Interestingly, the GAL administration to rats with acute colitis caused by TNBS resulted in a reduction in mucosal damage. It was also noted that treatment with galanin apparently reduced diarrhea in the tested animals. The antiinflammatory effects of galanin have also led to a decrease in neutrophil infiltration in the colon and a decrease in the level of TNF-α and expression of inducible NOS (Talero et al., 2006). Similar galanin activity was found in chronic TNBS-induced colitis, but the impairment of neutrophil function, as well as the decrease in TNF-α level, was less pronounced (Talero et al., 2007). This may suggest that the antiinflammatory effect of galanin is dependent on the phase of inflammation.

It has been reported that enteric nerve fibers simultaneously contain galanin, VIP and nNOS (Talero et al., 2007). In the digestive tract, receptors for neuroregulatory substances such as VIP or nitric oxide are found on the surface of immune cells (macrophages, lymphocytes, monocytes and granulocytes) (Maunder, 2000).

Nitric oxide produced by the neuronal nitric oxide synthase enzyme is the most important inhibitory nonadrenergic, noncholinergic (NANC) neurotransmitter (Sanders and Ward, 2019). In the ENS, only a small percentage of all submucosal neurons are nNOS-positive. In myenteric plexuses, the presence of nNOS is significant (Bódi et al., 2019). The presented results show the same relationship.

The action of pathological factors caused a change in the level of enteric nNOS expression. An increased number of mucosal NOS-IR nerve fibers were found in the duodenum, jejunum and colon of dogs with irritable bowel disease (IBD) (Rychlik et al., 2017). Similarly, in ulcerative colitis and Crohn’s disease, nNOS activity and NO production were increased in rectal mucosa (Ljung et al., 2006).

The reason for the involvement of NOS-positive neurons in enteroneuropathies may be the potentially damaging effects of a free radical – nitric oxide. Inflammation recruits immune cells to ganglia that release cytokines. Many cytokines and cell damage products, such as histamine, prostaglandins and interleukins, depolarize enteric neurons, resulting in increased intracellular neuronal Ca^2+^. Nitric oxide synthase is a Ca^2+^ dependent enzyme. Increased Ca^2+^ concentration activates NOS, which causes excessive production of NO (Rivera et al., 2011).

The current reports indicated that NO is involved in the inhibition of circular tone and motility of the inflammatory region of the gastrointestinal tract. Furthermore, Studies by Onori et al. indicate that active substances such as interleukin 1 (IL-1), tumor necrosis factor α (TNFα) and interferon γ (INFγ), which are extremely important in the inflammatory process of severe colitis, induce nitric oxide production (Onori et al., 2005). The increase in expression of this neurotransmitter may also suggest its participation in the development of drug-induced duodenal inflammation.

In the enteric nervous system, VIP and NO perform different functions in response to ongoing inflammation. The antiinflammatory effect of VIP has already been proven using various research models (Gonzalez-Rey et al., 2007; Olson et al., 2015; Makowska, 2018).

According to studies by Takeuchi et al., VIP and PACAP (both neurotransmitters belong to the same family of peptides) stimulate the duodenal secretion of bicarbonates (Takeuchi et al., 1998). Interestingly, VIP inhibits the production of proinflammatory cytokines and inflammatory mediators such as IL-6, TNF-α, IL-12 and chemokines (Delgado et al., 2003b). It is additionally synthesized by type 2 (Th2) T lymphocytes. Therefore, many authors view VIP as a Th2 cytokine (Delgado and Ganea, 2001).

VIP is involved in the differentiation of T helper cells toward the Th2 phenotype and regulates their secretory functions. It also stimulates the synthesis of neurotrophic compounds (for example, a brain-derived neurotrophic factor), suggesting its participation in the recruitment of new neurons (Quintana et al., 2006). In addition, it is an inhibitor of the proinflammatory effects of macrophages (Leceta et al., 2000).

The available literature data indicate that the immunohistochemical results regarding the effect of inflammation on VIPergic neurons are divergent. The effect of indomethacin on the duodenal wall caused a disturbance of neuronal homeostasis which, consequently, led to an increase in VIP expression in enteric plexuses. Similar observations were noted in the porcine duodenum with T-2 toxin poisoning (Makowska et al., 2018) and in the stomach of a pig after acrylamide administration (Palus et al., 2018). On the other hand, there are many reports indicating that inflammation has suppressed neural VIP expression (Sanovic et al., 1999; Boyer et al., 2005).

In low-grade inflammation, proinflammatory cytokines such as TNF-α and IL-1β inhibit intestinal smooth muscle contractility, but the spontaneous release of VIP from VIP-positive neurons opposes this effect. In high-grade inflammation, inflammatory mediators significantly inhibit VIP release from enteric cells (Shi and Sarna, 2009).

Data obtained in recent years indicate the antiinflammatory effect of PACAP. VIP and PACAP are highly homologous. As suggested from their structural similarity, these peptides bind to each other’s receptors, but with different affinity (Delgado et al., 2003b). An increase in PACAP as well as VIP expression is observed in enteric neurons in many experimental models such as naproxen treatment (Czajkowska et al., 2020) and duodenal ulcer model (Reglodi et al., 2018).

PACAP is a common compound whose expression has been demonstrated in nerve cells and in many immune cell populations. Recent studies have shown the expression and distribution of VIP/PACAP receptors in thymocytes, peripheral T and B lymphocytes, mouse macrophages and human monocytes (Delgado et al., 2003b). The expression of PACAP-preferring receptor PAC1 has been only noted in peripheral macrophages and monocytes. Macrophage stimulation is one of the main modes of PACAP activity. This leads to direct inhibition of proinflammatory cytokine production (TNF-α, IL-6, IL-12). An increase in IL-10 synthesis (a strong antiinflammatory result) has also been noted (Delgado et al., 1999). The antiinflammatory effect of PACAP is also revealed by inhibiting IL-2 production and T cell proliferation. PACAP is responsible for inhibiting Th1 response and is involved in generating Th2 cell memory (Delgado et al., 2003a).

The protective effect of PACAP has been described in many pathological processes in the gastrointestinal tract, such as colitis caused by dextran sulfate (Azuma et al., 2008) or intestinal ischemia (Ferencz et al., 2010). Interestingly, it has been documented that PACAP (mainly *via* the PAC1 receptor) is involved in the process of neuronal proliferation and also affects the axonal regeneration and the development of the nervous system (Gonkowski and Całka, 2012). Hence, it is tempting to speculate that the increase in PACAP levels obtained in this experiment is associated with the protective role of the peptide during ongoing drug changes in the duodenum.

Research in recent years has focused on CART function within the central nervous system. The share of peptide in feeding behavior has been demonstrated. CART mRNA expression depends on the concentration of leptin, which transmits a satiety signal to the hypothalamus (Kristensen et al., 1998). Injecting CART fragments into the cerebral ventricles results in a decrease in food intake. Nutritional behavior is inhibited and NPY’s effect on ingestion is blocked (Kristensen et al., 1998; Larsen et al., 2000). Unfortunately, information on CART expression in the gastrointestinal tract, both physiologically and as a result of the co-occurrence of inflammatory lesions, is insufficient. As it has been previously reported, CART-IR neuronal structures were observed in all layers of the intestinal wall in the pig (Wierup et al., 2007), the rat (Ekblad et al., 2003) and the guinea pig (Ellis and Mawe, 2003).

According to literature data, CART may have an inhibitory effect on gastric secretion. It has also been shown that the peptide regulates the smooth muscle activity of both the stomach and intestines. In the large intestine, CART has a stimulating effect on muscle cells, while in the stomach an inverse relationship has been noted (Okumura et al., 2000; Tebbe et al., 2004).

Analysis of literature data on the replacement of CART expression in enteric plexuses may suggest the participation of the peptide in neurotrophic and neuroprotective processes, as well as in neuronal regenerative mechanisms. In the central nervous system, CART increases neuronal survival, suggesting that the peptide has neuroprotective properties. According to Ekblad et al. (2003), an increase in CART expression in enteric neurons was noted in the atrophic rat intestine. This may suggest that CART is an important factor involved in the survival of nerve cells and/or muscle layer cells. In addition, the authors showed that under stress conditions or neuronal damage, CART has a neurotrophic effect (Ekblad et al., 2003; Ekblad, 2006).

In summary, this experiment revealed that indomethacin supplementation induces a reaction of porcine duodenum ENS neurons, expressed as an increased number of GAL, VIP, CART, SP, nNOS, and PACAP-like immunoreactive neurons. The observed changes may be associated with adaptive and neuroprotective changes of enteric neurons in response to indomethacin or may be due to inflammation that may accompany drug supplementation. In addition, this is evidence that in the presence of existing inflammation, enteric neurons are characterized by neuroplasticity by showing altered expression of neurotransmitters. Due to the widespread analgesic treatment with indomethacin, this study may be the starting point for further pharmacological and clinical studies to ensure the safety of pain control.

## Data Availability Statement

All datasets presented in this study are included in the article/supplementary material.

## Ethics Statement

The animal study was reviewed and approved by Local Ethical Committee in Olsztyn (decision no. 54/2017 from 25 July 2017).

## Author Contributions

Conceptualization: MC and JC. Methodology: MC. Formal analysis: JC. Investigation: MC and JC. Writing–original draft preparation: MC. Writing–review and editing: MC and JC. Visualization: MC. Supervision: JC. Project administration: MC and JC. Funding acquisition: MC. All authors contributed to the article and approved the submitted version.

## Funding

This study was supported by the National Science Centre - grant no. 2018/29/N/NZ4/00348 (cost of conducting the experiment). Project financially co-supported by Minister of Science and Higher Education in the framework of the program entitled “Regional initiative of Excellence” for the years 2019–2022, Project No. 010/RID/2018/19, amount of funding 12.000.000 PLN (the cost of publishing the article).

## Conflict of Interest

The authors declare that the research was conducted in the absence of any commercial or financial relationships that could be construed as a potential conflict of interest.
